# The impact of leadership interventions on neonatal care: a systematic review of current literature

**DOI:** 10.1007/s00431-024-05968-8

**Published:** 2025-01-11

**Authors:** Flavia Beccia, Maria Rosaria Gualano, Gianluca Fevola, Emanuele Capogna, Chiara Scarfagna, Michele Bonacquisti, Walter Ricciardi

**Affiliations:** 1 Section of Hygiene, University Department of Life Sciences and Public Health, Università Cattolica del Sacro Cuore, Rome, Italy; 2 UniCamillus - Saint Camillus International University of Health and Medical Sciences, Rome, Italy; 3 Leadership Research Center, Università Cattolica del Sacro Cuore-Campus Di Roma, Rome, Italy

**Keywords:** Leadership, Systematic review, Neonatal care

## Abstract

Effective leadership is essential in neonatal intensive care units (NICUs), where complex, high-stakes environments require coordinated multidisciplinary teamwork. Strong leadership improves clinical outcomes, team performance, and staff well-being. This systematic review assesses various leadership models and interventions in NICUs to identify best practices and areas for future research. A systematic search was conducted on PubMed, Web of Science, and Scopus, covering studies published from 2010 to October 2024. Articles were screened using the PRISMA guidelines, and inclusion criteria focused on primary studies in NICU settings evaluating leadership interventions. Data extraction and quality assessment were performed using the Newcastle–Ottawa Scale. Nine studies from diverse countries and research designs were included. Leadership interventions varied from simulation-based training programs to co-leadership models. High-fidelity simulation boot camps significantly improved self-perceived skills, teamwork, and leadership confidence among trainees. While most studies reported positive impacts on team performance and patient safety, one large-scale quality improvement program showed no significant improvement in clinical outcomes for very-low-birth-weight infants.

*Conclusion*: The findings emphasize that leadership interventions, including structured training and co-leadership, enhance team dynamics and clinical outcomes in NICUs. However, variability in study designs and reliance on self-reported data highlight the need for standardized evaluation methods. Future research should focus on long-term impacts, cross-context comparisons, and refining leadership frameworks to address the unique challenges of NICU settings. Promoting effective leadership not only improves patient care but also fosters a resilient and collaborative work environment.

**Table:** 

**What is known:** *• Leadership is crucial in NICUs, where complex, high-stakes environments demand coordinated, multidisciplinary teamwork. Strong leadership enhances clinical outcomes, team performance, and staff well-being.* *• No systematic review of leadership interventions in neonatal care has been conducted to date.*
**What is new:** *• Recent studies highlight a range of tools, including simulation-based training programs and co-leadership models.* *• High-fidelity simulations have been shown to significantly improve participants' self-perceived skills, teamwork, and leadership confidence.*

**What is known:**

*• Leadership is crucial in NICUs, where complex, high-stakes environments demand coordinated, multidisciplinary teamwork. Strong leadership enhances clinical outcomes, team performance, and staff well-being.*

*• No systematic review of leadership interventions in neonatal care has been conducted to date.*

**What is new:**

*• Recent studies highlight a range of tools, including simulation-based training programs and co-leadership models.*

*• High-fidelity simulations have been shown to significantly improve participants' self-perceived skills, teamwork, and leadership confidence.*

## Introduction

Effective leadership is a cornerstone of excellence in healthcare, significantly influencing patient outcomes, staff well-being, and the overall quality of care [1]. In medicine, leadership extends beyond administrative roles; it encompasses guiding clinical teams, fostering collaboration, and ensuring patient safety. Strong leadership is associated with enhanced clinical performance, reduced errors, and improved organizational culture [2, 3].

Neonatology, particularly in neonatal intensive care units (NICUs), presents unique challenges that underscore the importance of leadership. NICUs operate with high-stakes scenarios involving fragile patients, necessitating precise coordination among multidisciplinary teams, including nurses, physicians, and support staff [4]. Leadership in this setting is critical not only for clinical decision-making but also for managing complex team dynamics and maintaining a psychologically safe environment where staff feel valued and empowered to voice concerns or share insights [5].

A particular challenge in NICU leadership is managing “fluid teams,” where members frequently change due to shifts or the need for specialized input. This dynamic environment requires adaptable leadership that promotes consistency, clear communication, and cohesive teamwork. Effective leaders in NICUs must also navigate hierarchical structures while fostering an inclusive culture that encourages interprofessional cooperation. This is crucial for managing stress and preventing burnout, common issues in intensive care settings where the emotional toll can be significant [6].

Given these complexities, understanding and improving leadership within neonatology is essential for enhancing both clinical outcomes and team performance. Therefore, this systematic review aims to assess existing leadership adoption and models in NICUs, and for which aims the leadership is investigated. By synthesizing current evidence, this systematic review seeks to identify best practices and areas for future research, ultimately contributing to the development of robust leadership frameworks tailored to the unique needs of neonatal care.

## Methods

### Search strategy and eligibility criteria

This systematic review adhered to the Preferred Reporting Items for Systematic Reviews and Meta-Analyses (PRISMA) guidelines [7]. The database search was conducted and updated in October 2024, covering all articles published up to October 1, 2024, with a starting publication date of 2010. Searches were performed across three databases: PubMed, Web of Science, and Scopus, using the query: *(leadership AND (neonatology OR neonatal OR newborn OR child OR paediatric OR perinatal OR infant))*.

The query was adjusted for compatibility with Scopus and Web of Science.

After retrieving articles from the selected databases, duplicates were removed, and titles and abstracts were screened using the Rayyan platform [https://www.rayyan.ai/]. This tool enabled double-blind screening by researchers to minimize selection bias. Any conflicts during screening were resolved through internal discussion. To account for variability in terminology, the search query was not restricted to specifically “neonatal” settings.

### Inclusion and exclusion criteria

Articles written in English language that assessed leadership models in the neonatal setting were included in the review. Articles written in another language or that did not measure or assess the topic in the specific setting were excluded from the systematic review. Only primary studies were included.

### Data extraction and synthesis

Data extraction was performed by one author after the full-text inclusion was confirmed through consensus or conflict resolution. Extracted data were recorded in an Excel sheet and included the following details: first author, year, country, study type, population, setting, objective, main findings, and whether the leadership evaluation change was statistically significant. Results were synthesized and presented qualitatively.

### Quality assessment

The methodological quality of all included studies was evaluated using the Newcastle–Ottawa Scale [8].

## Results

**Fig. 1:**
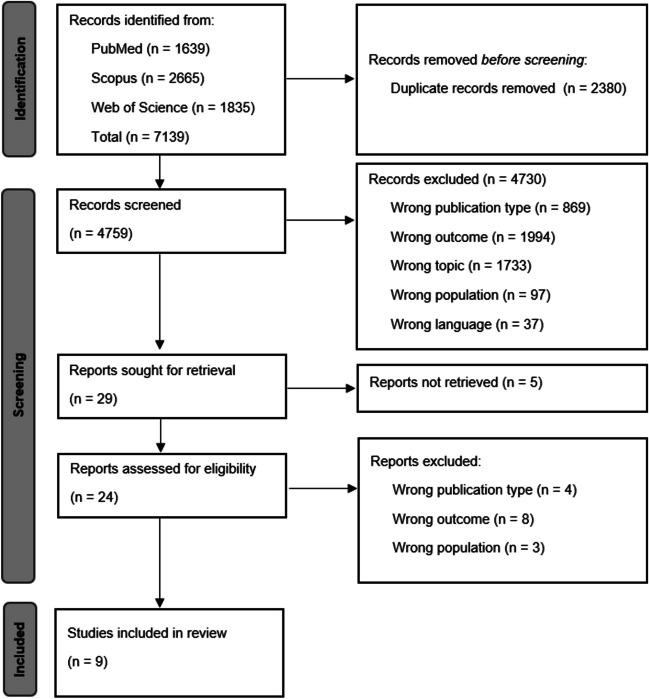
PRISMA flowchart

The initial search across three databases (PubMed, ISI Web of Knowledge, and Scopus) yielded 7139 records. After duplicate removal, five researchers independently screened the remaining entries, resolving any disagreements through internal discussion. Ultimately, nine studies were included in the systematic review (see Fig. 1: PRISMA flowchart) [9–17].

Detailed information about the included articles is presented in Table 1. All studies were assessed using the Newcastle–Ottawa Scale and demonstrated at least satisfactory methodological quality (scores ≥ 6). The articles were published between 2013 and 2024, with most studies conducted in the USA (*n* = 5, 55.6%) [14–18], followed by one study each from Japan [10], Germany [13], Zimbabwe [12], and the UK [9].

**Table 1:** Detailed information about the included articles

First author, year [Ref]	Country	Study type	Population	Setting	Objective	Main findings	Statistically significant change on leadership evaluation
Kannan Loganathan P., 2024 [9]	UK	Cross-over randomized pilot study	24 volunteer pediatric trainees, nurse practitioners, and neonatal nurses	NICUs simulation rooms	Evaluate team performance, behavioral skills, and individual workload with and without dedicated team leader	Participants without a team leader showed more fixations on manikins and monitors, though not significantly. Team performance, behavioral skills, and workload did not differ significantly. Physical demand was significantly higher without a team leader. All teams preferred having a dedicated leader	Yes
Nishida T, 2024 [10]	Japan	Cluster-randomized clinical trial	3435 infants	NICUs (in 40 hospitals)	To assess a comprehensive quality improvement program on outcomes in very-low-birth-weight (VLBW) infants. The primary outcome was survival without neurological impairment at 3 years of age	There were no significant differences in gestational weeks, birth weights, survival rates without neurological impairment, or mortalities at NICU discharge between the groups. The intervention group showed clinically significant improvements, including lower rates of sepsis, adrenal insufficiency, and anemia-related transfusions, along with a shorter time to full enteral feeding	No
Williams S, 2018 [18]	USA	Prospective, observational study	10 incoming neonatology fellows in 2016 and 2017	NICUs simulation rooms	To assess self-perceived skill confidence, teamwork, and team leadership Outcome measured on 6 categories: team leader in code, team leader in delivery room, make medical decisions, perform intubation, place chest tube, place umbilical catheter	A 1-day high-fidelity simulation boot camp significantly improved self-perceived skill confidence, teamwork, and leadership in first-year neonatal-perinatal medicine fellows	Yes
Ngwenya S, 2017 [12]	Zimbabwe	Retrospective cohort study	Neonatology ward health professionals 6 months prior to the implementation date (January 2016) and 12 months after the implementation date	NICU, labor ward in Mpilo Central Hospital	To assess fresh full-term stillbirths (> 37 weeks gestation) occurring during the intrapartum stage of labor to see reduction in numbers after the leadership and accountability measures put in place	There was a statistically significant reduction in the number of fresh full-term intrapartum stillbirths after the introduction of the measures. There was a statistical difference before and after implementation of the changes There was a reduction in the time it took to perform an emergency caesarean section	Yes
Kuntz L, 2020 [13]	Germany	Cross-sectional study	70 NICUs staff. Each NICU with a co-leadership dyad that comprises the leading nurse and the leading physician. Overall, the team sample encompasses responses from 1182 nurses and 440 physicians	NICUs	To assess the degree to which co-leaders share goals in general fosters a safety climate by pronouncing norms of interprofessional cooperation as a behavioral standard for the team members’ interactions	Analyses indicate that the extent of goal-sharing between nurse-physician co-leaders covaries with the safety climate in NICUs, partially mediated by interprofessional cooperation norms among team members	Yes
Colacchio K, 2012 [14]	USA	Prospective, observational study	176 neonatal attendings, fellows, nursing leadership and staff, nurse practitioners, physician assistants, respiratory therapists, patient care associates, and business administrators employed between October and November 2010	NICUs or simulation rooms	Develop a curriculum, adapted from TeamSTEPPS™. Measure Teamwork Attitudes and feedback survey	176 employees from various disciplines received teamwork training in 25 multidisciplinary sessions held over 6 weeks. 164 TAQs were analyzed. Physicians perceived teamwork behaviors to be a 4.49/5 vs. 4.44/5 (practitioners), 4.40/5 (nurses), and 4.25/5 (other disciplines)	NR
Sawyer T, 2013 [15]	USA	Prospective, observational study	42 physicians, nurses, and respiratory therapists	NICUs simulation rooms	To determine the impact of interprofessional Team Strategies and Tools to Enhance Performance and Patient Safety (TeamSTEPPS) training on teamwork skills during neonatal resuscitation	Significant improvements in teamwork skills were reported in team structure, leadership, situation monitoring, mutual support, and communication. The odds of a nurse challenging an incorrect medication dose from an attending neonatologist improved significantly	Yes
Cordero L, 2013 [16]	USA	Prospective, observational study	33 residents divided in 11 teams	NICUs simulation rooms	To evaluate the skills and team behavior of pediatric residents during resuscitation with a high-fidelity mannequin before and after a deliberate practice intervention. Skills were scored for technique and timeliness and team behaviors for communication, management, and leadership	Gaps in procedural skills noted during the first session were corrected. Timeliness for completion of skills remained below expectations. Improvements in team behaviors were noted Median communication and management scores improved significantly from the first to the second session Median leadership scores and median combined team scores improved significantly from the first to the second session	Yes
Heling AZ, 2020 [17]	USA	Prospective, observational study	99 residents	NICUs	To determine if a NICU resident delivery room (DR) skills educational curriculum is associated with changes in neonatal resuscitation team characteristics, including teamwork, communication, and leadership	Comparing behaviors at the beginning versus the end of a NICU block, residents demonstrated significant increased frequency of initiating leadership and maintaining leadership at low-risk, resident-attended DR resuscitations. Overall measurements of teamwork and communication were unchanged	Yes

The majority of the studies (*n* = 5, 55.6%) were prospective, observational investigations [14–18]. Leadership changes showed statistically significant effects on the primary outcomes in seven out of nine studies, while one study did not report these results [10].

Regarding target populations, all studies focused on healthcare professionals working in NICUs. The main settings were NICUs (*n* = 4, 44.4%) [10, 12, 13, 17] and NICU simulation rooms (*n* = 4, 44.4%), [9, 15, 16, 18], with one study incorporating both settings [14].

## Discussion

The studies reviewed emphasize the critical role of leadership in NICUs, highlighting both the complexity of leadership interventions and their impact on team performance and clinical outcomes. Loganathan’s pilot study, which compared teams with and without a dedicated leader, found no significant differences in terms of visual attention, behavioral skills, or self-reported workload. However, the team without a leader reported significantly higher physical demand and workload, suggesting that even in the absence of measurable improvements, the presence of a leader is essential for reducing team stress and improving dynamics [9].

The results reinforced the idea that leadership in NICUs goes beyond the individual leader. Kuntz and colleagues highlighted that shared understanding and vision between nurse-physician co-leaders, achieved through joint development programs, are crucial for fostering a safety climate and enhancing interprofessional cooperation [13]. This collaborative leadership model emphasizes teamwork and patient safety, a point echoed by other studies advocating for co-leadership and team training as means to improve communication and safety [19]. Transformational and self-leadership had a positive influence on job adaptivity [3].

Several studies demonstrated that structured teamwork training could significantly improve teamwork skills, leadership, communication, and mutual support within NICUs, enhancing team performance and patient safety [13, 15–17]. Leadership interventions such as simulation training, co-leadership models, and structured team training show promise in improving teamwork and clinical skills. However, the complexity of neonatal care necessitates continuous adaptation and evaluation of leadership strategies [10]. Future research should focus on assessing the long-term impacts of these interventions and exploring how leadership can be further integrated into training programs to address the unique challenges faced by NICU teams [20].

The findings of this systematic review fill a gap in the existing evidence and highlight the crucial role of leadership in NICUs. Previous research underscores that effective leadership enhances patient outcomes, improves staff morale, and fosters a culture of safety and collaboration [20]. Collaborative and inclusive leadership models reduce clinical errors and promote psychological safety within healthcare teams. These factors contribute to a supportive environment where healthcare professionals feel valued and empowered to voice concerns, a critical component in high-stress settings like NICUs.

In addition, Ngwenya’s study demonstrates that leadership focused on resource management and organizational strategies can have a substantial impact, especially in settings with limited resources [12]. However, Nishida’s cluster-randomized trial, which evaluated a comprehensive quality improvement program in NICUs across Japan, did not demonstrate significant improvements in clinical outcomes for very-low-birth-weight infants. Despite the extensive nature of the program, the lack of improvement may underscore the challenges of implementing large-scale interventions in the complex, high-risk environment of neonatal care [10].

### Strengths and limitations

This systematic review offers a thorough analysis of leadership interventions in diverse NICU settings, drawing from data across multiple countries and study designs. The inclusion of both observational and randomized studies strengthens the findings, providing a comprehensive view of how leadership impacts team performance and patient outcomes. The methodology employed, along with the broad search query, facilitated the retrieval of a wide range of relevant articles, ensuring a robust and inclusive collection of studies. These factors collectively enhance the reliability and generalizability of the review’s conclusions.

However, this review has some limitations. First, the heterogeneity of the included studies in terms of design, sample size, and intervention type may introduce variability that could affect the generalizability of the results. Second, while most studies reported statistically significant findings, a few did not, potentially indicating publication bias toward positive results. Additionally, differences in healthcare systems and cultural contexts across countries may influence the applicability of certain leadership models in different settings. Finally, the reliance on self-reported measures for evaluating leadership and teamwork performance in some studies may introduce response bias, limiting the objectivity of the findings.

### Future perspectives

Future research should focus on longitudinal studies to assess the long-term impact of leadership interventions in NICUs. Comparative studies across different healthcare systems could also provide valuable insights into contextual factors that influence leadership effectiveness. Moreover, exploring the role of co-leadership models and their impact on team dynamics and patient outcomes could offer practical solutions for managing “fluid teams”.

## Conclusion

This review highlights the pivotal role of leadership in enhancing team performance and clinical outcomes in NICUs. Structured leadership training programs and interventions, particularly those emphasizing collaboration and communication, have demonstrated significant benefits. Promoting effective leadership in NICUs not only improves the quality of care for vulnerable patients but also fosters a resilient and cohesive work environment.

## Data Availability

No datasets were generated or analysed during the current study.
